# Lessons From the Processing and Sharing of Public Data Sets for the Study of Structural Racism

**DOI:** 10.1093/geroni/igaf122.1477

**Published:** 2025-12-31

**Authors:** Erik Westlund, Boeun Kim, Sierra Grey-Coker, Karen Bandeen-Roche, Sarah Szanton

**Affiliations:** Johns Hopkins Bloomberg School of Public Health, Baltimore, Maryland, United States; University of Iowa, Iowa City, Iowa, United States; Johns Hopkins University, Baltimore, Maryland, United States; Johns Hopkins Center on Aging and Health, Baltimore, Maryland, United States; Johns Hopkins University, Baltimore, Maryland, United States

## Abstract

As part of a larger study of structural racism, we collected 50 publicly available data sets containing geographic measures. These data sets covered over 100 years of history, six geographic units, and nine domains of inquiry (civics, credit/income/wealth, education, employment, environment, healthcare, media/marketing, neighborhoods, and policing). Structured metadata about each data set were compiled and used to standardize data files with shared conventions, allowing analysts to combine data files to study structural racism. To allow researchers to assess the potential value of these data in relation to their areas of inquiry, we created dashboards that summarize key measures in each data set, including the geographic level of measurement, the years covered by the data, and the extent of missingness. This process made clear several problems researchers seeking to use public data files to study structural racism will face, such as recency bias, temporal and geographic mismatch of data sources, and data missingness. We also developed data transformation pipelines to process each data file to follow shared conventions that allow reliable linking of data files across spatial units and time periods. To help outside researchers to use these data in their own projects, we created a public repository of structured metadata about the form and content of data files, accompanied by tools to allow users to procure data files, process them, and analyze them. This effort provided insight into the challenges faced by researchers trying to follow best practices with respect to open science, particularly around data licenses and data custody.

